# Systemic Comorbidity in Children with Cataracts in Nigeria: Advocacy for Rubella Immunization

**DOI:** 10.1155/2015/927840

**Published:** 2015-08-17

**Authors:** Roseline Duke, Sidney Oparah, Adedayo Adio, Okon Eyo, Friday Odey

**Affiliations:** ^1^Department of Ophthalmology, University of Calabar Teaching Hospital, Calabar, Cross River State, Nigeria; ^2^Department of Internal Medicine, University of Calabar Teaching Hospital, Calabar, Cross River State, Nigeria; ^3^Department of Ophthalmology, University of Port Harcourt Teaching Hospital, Port Harcourt, Nigeria; ^4^Department of Community Medicine, University of Calabar Teaching Hospital, Calabar, Cross River State, Nigeria; ^5^Department of Pediatrics, University of Calabar Teaching Hospital, Calabar, Cross River State, Nigeria

## Abstract

*Background*. Congenital and developmental cataracts are leading causes of childhood blindness and severe visual impairment. They may be associated with systemic diseases including congenital heart diseases which are among the major causes of morbidity and mortality in childhood. The pattern of systemic comorbidities seen in children diagnosed with cataract in Calabar, Nigeria, was studied. *Methods*. A retrospective review was conducted on the children who had cataract surgery between 2011 and 2012. Diagnosis of the systemic condition was documented. *Results*. A total of 66 children were recruited for the study. Cardiac disease was seen in 26 children (39.9%), followed by delayed milestone in 16 (24.2%), intellectual disability in 14 (21.2%), deafness in 11 (16.7%), epilepsy in 4 (6.1%), and physical handicap in 3 (4.5%) of them. Clinically confirmed Congenital Rubella Syndrome was seen in 30 (45%) of the children. The pattern of CHD seen was as follows: patent ductus arteriosus in 16 (24.2%) followed by ventricular-septal defect in 5 (7.6%), atrial-septal defect in 3 (4.5%), and pulmonary stenosis in 2 (3%). *Conclusion*. Systemic comorbidities, especially cardiac anomalies, are common among children with cataract in Nigeria. Congenital Rubella Syndrome may be a prominent cause of childhood cataract in our environment. Routine immunization of school girls against rubella is advocated as a measure to mitigate this trend.

## 1. Introduction

Congenital and developmental cataracts are the primary causes of treatable childhood blindness and severe visual impairment in Nigeria. Oftentimes, it may be associated with systemic conditions, resulting in children with multiple disabilities [1]. Childhood cataract is difficult to manage. However, standard age related surgical procedures have been developed and best practices recommended for optimal visual outcome and management of the evolving visual development in these children. Moreover, children with multiple disabilities have the need for varying and complex management involving multiple professional medical teams to effect adequate and appropriate care of not only the ocular condition but also the systemic conditions.

Investigations into the etiology of congenital cataract in some developing countries such as India have suggested that at least 12% are from preventable causes [2], whereas up to 70% of the condition in children in the developed countries are of idopathic origin [3]. In Nigeria, the etiology of pediatric cataract is not well defined. Investigations into the etiology are not only expensive and challenging but also difficult to conduct. It invariably involves a wide array of biochemical, microbiological, and genetic studies.

If the incidence of congenital cataracts is to be reduced, research into its etiology is imperative. Proxy methods to ascertain etiology may be a first useful step in recommending prevention and control measures in the absence of any other data. Systemic co-morbid conditions occurring with congenital and developmental cataracts could highlight associations.

With the increasing number of paediatric cardiologists and the establishment of tertiary healthcare institutions in Nigeria with available echocardiography services, more cases of congenital heart disease (CHD) in children are being diagnosed [4]. CHD is a common cause of morbidity and mortality in children and it is a major public health concern [5]. One of the most significant public health causes of congenital cataract is rubella [6]. The link between congenital cataract and congenital heart defects as well as their association with rubella has long been established [7]. However, very few reports that establish this relationship have been documented in Nigeria. This study describes the pattern of systemic comorbid conditions, especially congenital heart defects in a population of Nigerian children undergoing cataract surgery.

## 2. Subjects and Method

This study was done in Calabar, the capital city of Cross River State (CRS), located in the oil rich Niger delta region of Nigeria. The Nigerian 2006 population census put the total population figures of Cross River State and Nigeria at around 2.9 million and 140 million, respectively. However, the recent population estimates, as those of 2011, state that the CRS population figure is about 3,337,517 and that of Nigeria is about 150 million [8]. The age-sex structure of the population shows a broad based pyramid which indicates that Nigeria's population is young, a scenario typical of developing countries with high fertility rates. As of 2013, women in Nigeria have a total fertility rate of 5.5 children. The proportion of children under the age of 15 years is around 46 percent, giving rise to the estimated pediatric populations of about 1.53 million and 69 million for Cross River State and Nigeria, respectively [8].

We conducted a retrospective review of children who had cataract surgery between January 2011 and December 2012. The children scheduled to have cataract surgery were accordingly referred for pediatric; neurological; ear, nose, and throat (ENT); audiometric measurement; and echocardiograph evaluations in the University of Calabar Teaching Hospital. Such multidisciplinary assessment was to define and confirm the presence of systemic conditions and the presence of identifiable CHD. The children were also evaluated with respect to fitness for the administration of general anesthesia. Data on the history of the child and demographic details such as age, gender, ocular, and systemic examination findings were collected. The type of CHD was ascertained by the cardiologist after interpreting the echo cardiogram result.

The case definition of “clinically confirmed” Congenital Rubella Syndrome (CRS) in the study includes any infant less than one year old who has at least two of the complications listed in (a) or one in (a) and one in (b) as below: (a) cataract, congenital glaucoma, congenital heart disease, loss of hearing, and pigmentary retinopathy; (b) purpura, splenomegaly, radiolucent bone disease, and jaundice that begins within 24 hours after birth [7].

Delayed developmental milestone was defined as any ongoing delay or multiple delays in reaching milestones.

The diagnosis of intellectual disability, formally termed mental retardation, was obtained from history and observation of adaptive behaviors in more than one hospital visit and defined as a disorder with onset during the developmental period associated with difficulty in functioning in daily life in areas such as communication, self-care, home living, social/interpersonal skills, self-direction, and academics, health, and safety.

Deafness was ascertained by an ENT surgeon and from the result of audiology when performed. “Hearing impairment” meant loss of sixty decibels or more in the better ear in the conversational range of frequencies [9]. Physical handicap was defined as any disability in locomotor function.

*Epilepsy*. It is any evidence of strange sensations, emotions, and behavior or sometimes convulsions, muscle spasms, and loss of consciousness seen or reported by the caregiver.

In this study, multiple systemic comorbidity is defined as the presence of more than one abnormal systemic condition or impairment in a child with cataract.

Analysis was performed using SPSS 22 (Chicago, Illinois). The Ethical Committee of the University of Calabar Teaching Hospital approved the study.

## 3. Results

A total of 66 children were reviewed, comprising 38 (57.6%) males and 28 (42.4%) females. The age of the participants ranged from 0.3 to 15 years, with a mean age of 5.1 ± 3.63 years.

Thirty-two (48.5%) of the children, comprising 19 (28.8%) males and 13 (19.7%) females, had coexisting systemic conditions, of whom sixteen (24.2%) presented with multiple systemic comorbidities. There was no gender difference in the occurrence of systemic comorbidities (*P* value > 0.7741). The children with systemic comorbidities were younger than those who did not have coexisting systemic conditions, with mean ages of 3.8 ± 3.36 and 6.2 ± 3.84, respectively (*P* value = 0.007).

The types and frequency of the occurrence of each systemic comorbid conditions are as shown in Table 1. Twenty-six (39.4%) of the children had cardiac anomalies, of which 16 (24.2%) were males and 10 (15.2%) were females.  Table 2 shows the types and frequencies of cardiac anomalies.

Clinically confirmed Congenital Rubella Syndrome (CRS) was seen in 30 (45.5%) of children.

## 4. Discussion

For every two children with congenital or developmental cataract seen in the study, one will have a systemic comorbid condition or impairment in addition to the cataract. This is similar to what was seen in another population [10]. Multiple comorbid conditions may often result in children who have multiple disabilities, the combination of which would require special educational needs such that these children cannot be accommodated in special education programs solely for one of the impairments. Furthermore, it was seen that children with systemic comorbidities were younger than those who did not have coexisting systemic conditions. This may be due to an early demise of children with coexisting systemic conditions, as a study has shown a relationship between morbidity and longevity [11]. The life expectancy and quality of life (QoL) of children with combined disorders need to be further investigated.

The pattern and frequency of CHD seen in this study appear to suggest that the main etiology of cataracts is rubella. This was confirmed by the presence and number of children seen in this study with clinically confirmed Congenital Rubella Syndrome. The pattern of CHD that we observed in our study is similar to what was seen years ago in the USA during the rubella epidemic [12]. Congenital rubella is a significant yet preventable cause of cataract in our population. Addressing the problem by sustained health education and the administration of an effective vaccine is recommended as a public health intervention. A successful MMR vaccination program would result in the prevention of the physical, emotional, and psychological trauma associated with prenatal rubella infection [13]. The rubella vaccination in school age girls is essential for this population, especially considering the high cost of treatment of cataract and managing the psychosocial challenges families encounter from an entirely preventable disease.

In Nigeria, rubella infection has not been given the necessary publicity and attention needed for its prevention and control. Such an attention is critical if rubella infection must be eradicated in women of child bearing age. There is no policy yet in Nigeria that makes rubella a notifiable disease, neither is there a national vaccination programme implemented for its control, let alone its eradication. The mumps-measles-rubella vaccination is not part of the routine immunization coverage for children as it is in the developed countries. However, it is noted that individual childhood vaccination is not the most important factor in bringing down the incidence of the infection as seen in the history, but it is rather an appropriate vaccine programme and good vaccine coverage in women of child bearing age.

In the global village of today's world, concerns have been raised by countries that have put in effort to address the challenges of rubella. Such countries are assumed to be at risk of rubella importation and resurgence of the disease if strong surveillance systems are not put in place by countries in the African continent that have no rubella immunization programme [14].

## 5. Conclusion

Systemic comorbidities, especially cardiac defects, are common in Nigerian children presenting with cataract. Presentation with childhood cataract in our environment should therefore raise the index of suspicion for rubella and the need for cardiac evaluation in such a child.

Congenital Rubella Syndrome is a prominent and clinically important cause of childhood cataract in our environment. Rubella vaccination in school age girls is strongly recommended as a preventive and control measure against this disease and its trend of comorbidities.

## Figures and Tables

**Table 1:** Types and frequency of occurrence of systemic comorbidities in children with cataract.

Systemic comorbidity	Frequency	Percentage (%)
Cardiac disease	26	39.4
Delayed milestones	16	24.2
Mental retardation	14	21.2
Deafness	11	16.7
Epilepsy	4	6.1
Physical handicap	3	4.5

**Table 2:** Types and frequencies of congenital heart defects.

Type of cardiac defect	Frequency	Percentage (%)
PDA	16	24.2
VSD	5	7.6
ASD	3	4.5
PS	2	3.0
None	40	60.6

Total	66	100

PDA = patent ductus arteriosus; VSD = ventricular-septal defect; ASD = atrial-septal defect; PS = pulmonary stenosis.
